# A systematic review and meta-analysis of the prevalence and risk of syphilis among blood donors in Thailand

**DOI:** 10.1038/s41598-025-94332-3

**Published:** 2025-03-18

**Authors:** Rujikorn Rattanatham, Wanida Mala, Kwuntida Uthaisar Kotepui, Frederick Ramirez Masangkay, Chutima Rattanawan, Supakanya Lasom, Kinley Wangdi, Manas Kotepui

**Affiliations:** 1 Medical Technology Program, Faculty of Science, Nakhon Phanom University, Nakhon Phanom, Thailand; 2 Department of Medical Technology, Faculty of Pharmacy, University of Santo Tomas, 1008 Manila, Philippines; 3 Department of Medical Science, Amnatcharoen Campus, Mahidol University, Amnatcharoen, Thailand; 4 Department of Medical Technology, School of Allied Health Sciences, University of Phayao, Phayao, Thailand; 5 HEAL Global Research Centre, Health Research Institute, Faculty of Health, University of Canberra, Bruce, ACT 2617 Australia; 6 National Centre for Epidemiology and Population Health, Australian National University, Acton, ACT 2601 Australia

**Keywords:** Syphilis, Blood donors, Prevalence, Thailand, Meta-analysis, Systematic review, Bacterial infection, Risk factors, Infectious diseases

## Abstract

Despite rising rates of sexually transmitted infections (STIs) in Thailand, including syphilis, data on its prevalence and associated risk factors among blood donors remain scarce. This systematic review and meta-analysis aimed to estimate the prevalence of syphilis and identify the key risk factors among blood donors in Thailand to inform targeted interventions for enhanced blood safety.The study protocol was registered in PROSPERO (CRD42024560215) and conducted following PRISMA guidelines. Comprehensive searches were performed across major databases, including ProQuest, Journals@Ovid, Embase, Scopus, PubMed, and MEDLINE, to identify relevant studies. A random-effects model was used to calculate the pooled prevalence and odds ratios (ORs) for syphilis risk factors. Heterogeneity was quantified using the *I²* statistic, and meta-regression and subgroup analyses were employed to explore potential sources of heterogeneity. Publication bias was assessed using funnel plots and Egger’s regression test. A total of 23 studies involving 1,142,910 blood donors were included. The pooled prevalence of syphilis among blood donors in Thailand was 0.42% (95% CI 0.27–0.66%, *I²*: 99.3%, number of infections: 6,173), with a decreasing trend over time. Male donors were significantly more likely to have syphilis (*P* < 0.0001; pooled OR: 1.76; 95% CI 1.53–2.03; *I²*: 34.2%, 651,019 participants). First-time donors also had an elevated risk of syphilis (*P* = 0.02; pooled OR: 2.02; 95% CI 1.10–3.70; *I²*: 94.0%, 215,245 participants). Additionally, the analysis revealed a significant association between increasing age and higher syphilis risk among blood donors. The systematic review and meta-analysis indicated that syphilis prevalence among blood donors in Thailand is declining; however, male and first-time donors, as well as older age groups, remain at higher risk. To enhance blood safety, targeted interventions are needed, including refining donor screening questionnaires, expanding educational campaigns for high-risk groups, and incorporating advanced screening technologies. Strengthening and regularly updating national screening policies will ensure the effective mitigation of syphilis transmission among blood donors in Thailand.

**Supplementary Information:**

The online version contains supplementary material available at 10.1038/s41598-025-94332-3.

## Introduction

Syphilis is an infectious disease caused by *Treponema pallidum* (*T. pallidum*), primarily transmitted through unprotected sexual contact and vertically from mother to fetus during gestation^1^. According to the World Health Organization (WHO), the global incidence of syphilis reached 7.1 million cases in 2020, with a prevalence of 8 million among adults aged 15–49 by 2022. In addition to sexual transmission, syphilis can also be transmitted through blood products and organ donation^2^. The prevalence of syphilis among blood donors varies globally, with rates of 28.4 per 100,000 donations in the United States (2020–2022)^3^, 0.14% in Bojonegoro, Indonesia (2020–2021)^4^, 0.33% in Jinan, China^5^, and 0.43% across China^6^. Southern China reported a higher prevalence of 370.1 per 100,000^7^, while Western Rajasthan, India, reported a prevalence of 1.02% in 2020^8^. In Brazzaville, Republic of Congo, the prevalence was lower at 0.7%, with a higher incidence among females aged 45–65^9^. Common risk factors include older age, lower education, and first-time donor status, with significant associations between syphilis and human immunodeficiency virus (HIV) infection in the United States^3^. In China, additional risk groups include females, those aged 35–44, farmers, and adults aged ≥ 45 years (621.8 per 100,000)^5–7^.

Although the prevalence of transfusion-transmissible infections (TTIs) among blood donors has been documented across various regions of Thailand, most studies have primarily focused on more common infections such as HIV, hepatitis B virus (HBV), and hepatitis C virus (HCV)^10–15^. In line with guidelines from the WHO^16^ and the National Blood Centre, Thai Red Cross Society (NBC-TRCS)^17^, the screening of all donated blood units for HIV, HBV, HCV, and syphilis is mandatory. However, there is limited evidence regarding the transmission of syphilis through blood donations in Thailand. To address this gap, a systematic review and meta-analysis was conducted to estimate the prevalence and identify risk factors for syphilis among blood donors in Thailand. The findings from this study will provide current evidence and insights to evaluate and enhance existing interventions aimed at improving blood safety in the Thai context.

## Methods

### Protocol registrations

The protocol for the systematic review was registered in PROSPERO (CRD42024560215). The systematic review and meta-analysis were reported following the Preferred Reporting Items for Systematic Reviews and Meta-Analyses (PRISMA) guidelines^18^.

### Systematic review questions

The primary aim of the systematic review was to determine the pooled prevalence of syphilis among blood donors in Thailand. The secondary aim was to assess the pooled risk of syphilis in this population. The PECO framework^19^ was used to formulate the research questions for this systematic review and meta-analysis. The target population (P) was blood donors; the exposure of interest (E) and the comparison group (C) included factors such as gender (male vs. female), frequency of donations (first-time vs. repeated), and age of blood donors (17–20 years vs. 21–30 years vs. 31–40 years vs. 41–60 years). The measured outcomes (O) were the pooled prevalence and the risk of syphilis among blood donors.

### Eligibility criteria

The eligibility criteria for the systematic review were designed to include studies that focused on the prevalence and risk factors of syphilis among blood donors in Thailand. Inclusion criteria required studies to involve blood donors as the target population specifically and to report relevant data on syphilis prevalence or associated risk factors. Studies employing various designs, such as retrospective descriptive, cross-sectional, and case-control studies, were considered. Also, only studies written in English or Thai language were eligible. Exclusion criteria included conference abstracts, review articles, and studies lacking specific information on syphilis in blood donors. Studies that did not focus on the relevant participants, outcomes, or study design were also excluded.

### Search strategy

The search strategy for this systematic review was conducted across several major databases, including ProQuest, Journals@Ovid, Embase, Scopus, PubMed, and MEDLINE. The search terms were tailored to capture studies related to syphilis, blood donors, and the specific context of Thailand, with the main terms including ‘Syphilis,’ ‘Blood Donors,’ and ‘Asia.’ Details of the search strategy in each database are listed in Table S1. The search was conducted from inception to June 20, 2024, with no restrictions on the publication date of the selected studies. To further ensure inclusiveness, the search was expanded beyond these databases. An additional search was conducted in Google Scholar^20^ and the Thai Journal Citation Index (TCI) on 20 June 2024. The Google Scholar search used the terms ‘Syphilis AND Blood Donors AND Thailand,’ while the TCI search employed the Thai-language terms ‘ซิฟิลิส OR Syphilis.’ The time frame for these searches was consistent with the other database searches, and all relevant results were reviewed. Reference lists of the included studies were also reviewed to maximize the identification of relevant studies.

### Study selection and data extraction

The study selection and citation management process were conducted using EndNote version 21.0 (Philadelphia, PA). Initially, all identified records from major databases were screened based on their titles and abstracts to exclude irrelevant studies. The full texts of potentially eligible studies were retrieved and assessed against the predefined inclusion and exclusion criteria. Studies that met the eligibility criteria were selected for inclusion in the systematic review. Data were extracted on key information such as study characteristics (publication year, study design, study location), participant demographics (male percentage, age range, nationality, blood group, donation frequency, collection sites), diagnostic methods for syphilis (treponemal/nontreponemal methods), and outcomes (number of syphilis infections). Relevant data related to the prevalence and risk factors of syphilis among blood donors was then entered into an Excel spreadsheet (Microsoft Excel 2021, Microsoft Corporation, USA). Two independent authors (RR, MK) performed the study selection, and disagreements were resolved through discussion to reach a consensus. Data extraction was conducted by two independent authors (RR, WM), with any disagreements resolved through review by a third author (MK).

### Risk of bias assessment

The Joanna Briggs Institute (JBI) critical appraisal tool was used to assess the risk of bias in the included studies^21^. Each tool is designed to address crucial aspects specific to each study type. Two independent authors (RR, KUK) performed the risk of bias assessment, and disagreements were resolved through a third author (MK) review.

### Data synthesis and statistical analysis

The meta-analysis of the pooled prevalence and odds ratio (OR) of syphilis among blood donors was conducted using a random-effects model to account for the variability and heterogeneity of outcomes across the included studies^22^. A fixed-effects model was also employed as a parallel analysis to assess the consistency of the findings, providing a robust check for the meta-analysis results. Heterogeneity was quantified using the *I²* statistic, with values greater than 50% indicating significant heterogeneity among the studies^23^. Each study is represented in forest plots by a square proportional to its weight, with horizontal lines indicating the 95% confidence intervals (CIs). Blue squares represent the study-specific estimates, and the vertical dashed line represents the overall effect estimate. Pooled prevalence and pooled ORs are shown using fixed-effects and random-effects models. For outlier detection, a leave-one-out sensitivity analysis was performed, where each study was sequentially excluded to evaluate its impact on the overall results^24^. Potential publication bias was assessed using visual inspection of the funnel plot and Egger’s regression test, provided that at least ten studies were included in the meta-analysis^25^. To explore potential sources of heterogeneity, meta-regression analysis assessed the impact of various study-level covariates, including publication year, study design, study location, percentage of male donors, first-time donation rates, onsite donation percentages, and the method used for syphilis detection. Subgroup analyses were also performed to determine whether specific factors, such as publication year, study design, study location, and syphilis detection method, contributed to variations in syphilis prevalence and OR across the studies. These analyses provided a deeper understanding of the factors influencing the study outcomes. All statistical analyses were conducted using RStudio (Version: 2024.04.2 + 764), which facilitated the implementation of meta-analysis procedures and statistical tests^26^.

## Results

### Search results

**Fig. 1:**
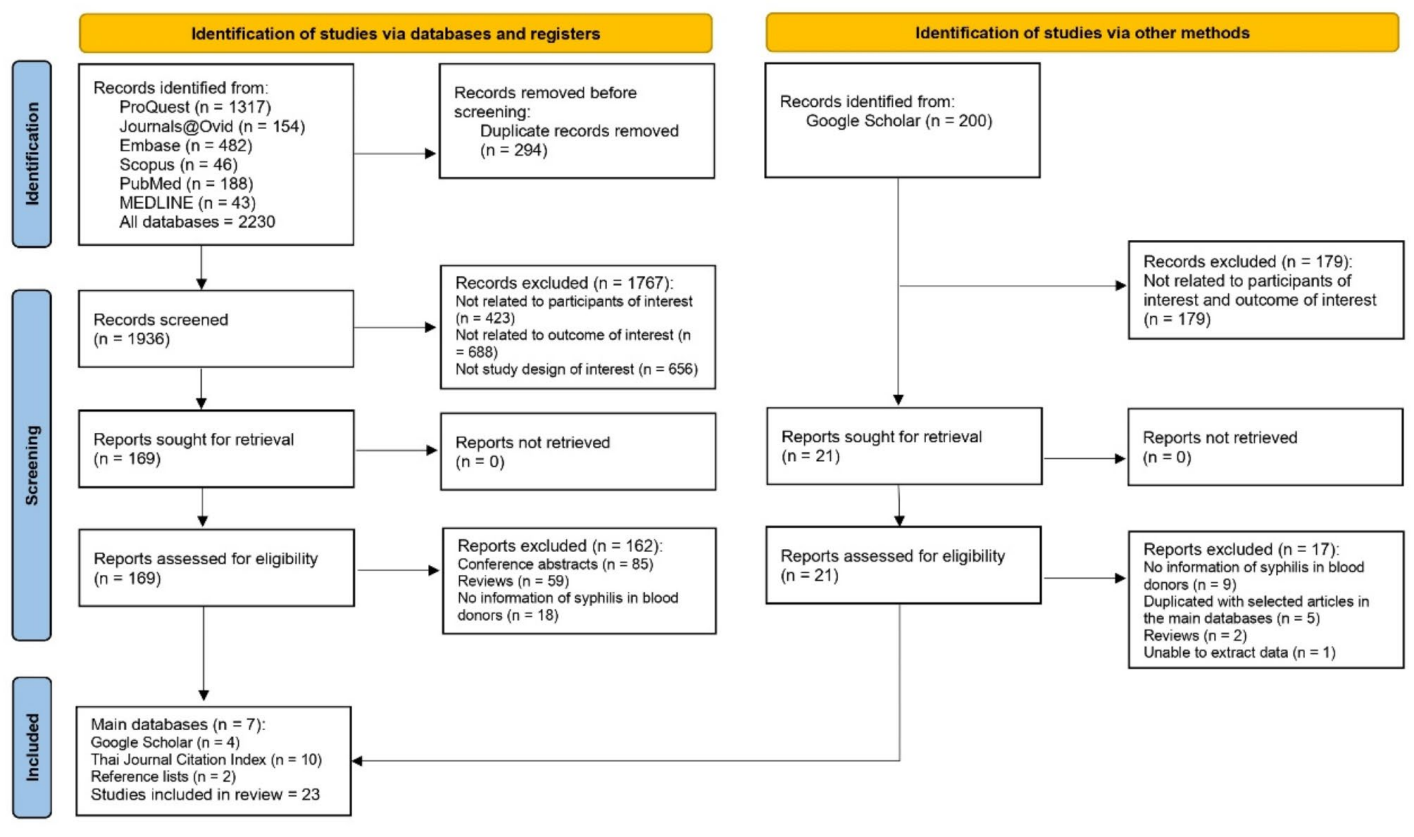
Study flow diagram.

Initially, 2,230 records were identified from the main databases. Following the removal of 294 duplicate records, 1,936 were screened. Of these, 1,767 records were excluded due to non-relevant participants, outcomes, or study design of interest. Subsequently, 169 articles were sought for full-text retrieval, all of which were successfully retrieved. During the eligibility assessment, 162 articles were excluded, primarily from conference abstracts, reviews, and studies lacking relevant information on syphilis in blood donors. Of the seven studies identified as eligible from the main databases, an additional four were found by searching 200 articles in Google Scholar. Furthermore, ten articles in Thai were identified from the TCI. A review of reference lists from the main databases and TCI yielded two more relevant articles. Twenty-three studies were included in the final review (Fig. 1).

### Characteristics of included studies

Table 1 summarizes the characteristics of the 23 studies included in the review. The studies span several decades, with 26.1% published before 2000, 17.4% between 2000 and 2009, 34.8% between 2010 and 2019, and 21.7% from 2020 to 2024. Most of the studies (82.6%) utilized a retrospective descriptive design, while a smaller portion consisted of cross-sectional (13.0%) and retrospective case-control (4.4%) studies. Geographically, the research was distributed across various regions of Thailand, with Central and Northern Thailand each contributing 21.7% of the studies, followed by Lower Northern Thailand (17.4%), Northeastern Thailand (13.0%), and Southern, Western, and Eastern Thailand each representing 8.7%. Regarding diagnostic methods for syphilis, nearly half of the studies (47.8%) used nontreponemal serology, 26.1% used treponemal serology, 21.7% employed both, and 4.35% did not specify the diagnostic method used. Details of studies included in the systematic review and meta-analysis are shown in Table S2.

**Table 1:** Summary characteristics of included studies.

Characteristics	*N*. (23 studies)	%
Publication year		
Before 2000	6	26.1
2000–2009	4	17.4
2010–2019	8	34.8
2020–2024	5	21.7
Study designs		
Retrospective descriptive study	19	82.6
Cross-sectional study	3	13.0
Retrospective study, case-control	1	4.35
Study areas		
Central Thailand	5	21.7
Northern Thailand	5	21.7
Lower northern Thailand	4	17.4
Northeastern Thailand	3	13.0
Southern Thailand	2	8.70
Western Thailand	2	8.70
Eastern Thailand	2	8.70
Diagnostic method for syphilis		
Serology (nontreponemal)	11	47.8
Serology (treponemal)	6	26.1
Serology (treponemal/nontreponemal)	5	21.7
Not specified	1	4.35

### Risk of bias across included studies

In the cross-sectional studies^27–29^, all met the key criteria, including valid and reliable measurement of both exposure and outcomes (Table S3). However, some were limited regarding unclear inclusion criteria^27^ and a lack of attention to confounding factors^27,29^. All studies for prevalence studies^10–15,30–43^ met the key criteria, including appropriately sampled study participants and using valid methods for identifying the condition. However, some were limited regarding the unclear adequacy of the sample size^15,33,36,40,42^, insufficient detail on study subjects and settings^11,37,41^, and unclear use of appropriate statistical analysis^13,31,33,36,37,41^. None of the studies included in the systematic review were excluded based on the JBI checklist criteria.

### Prevalence of syphilis among blood donors in Thailand

**Fig. 2:**
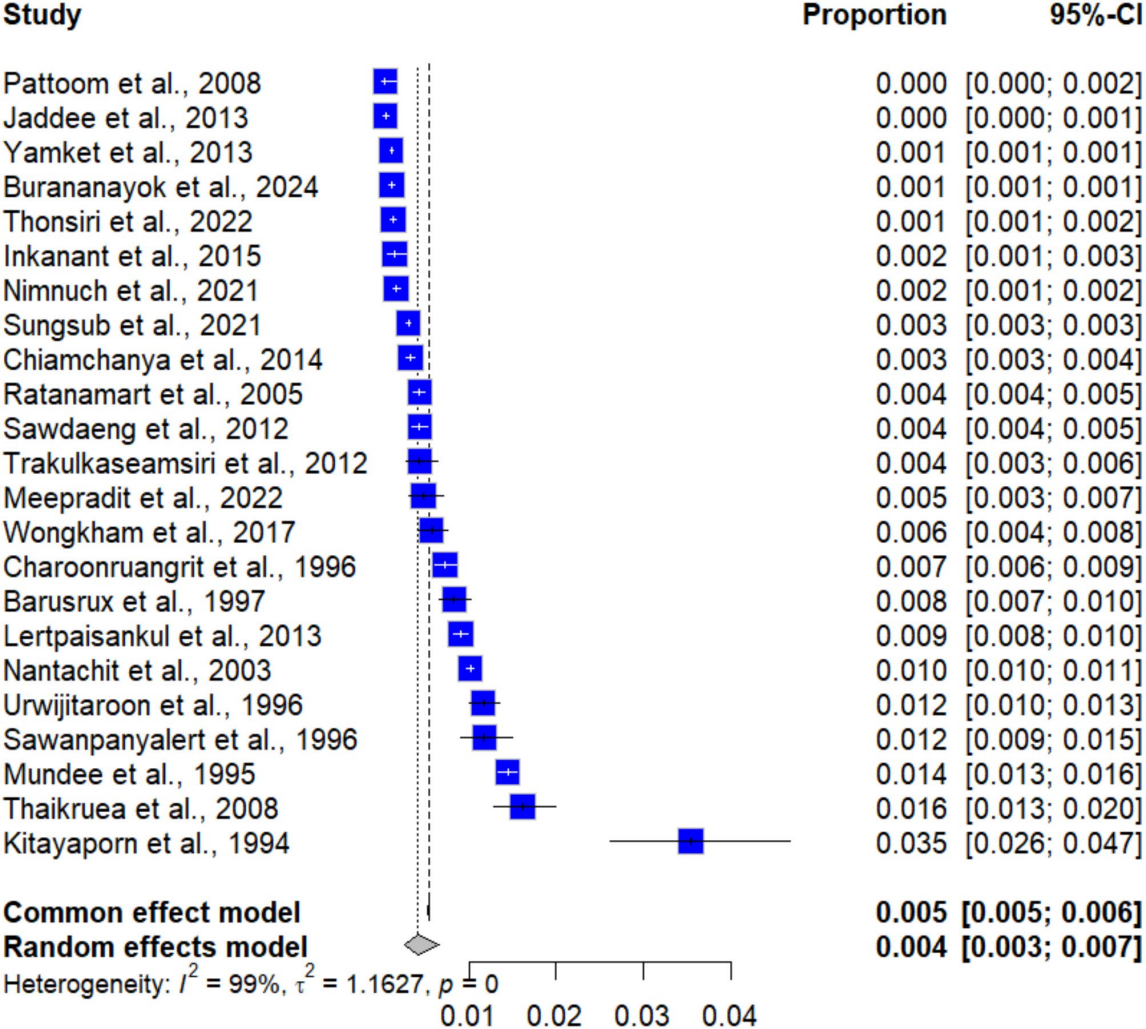
Forest plot showing the prevalence of syphilis among blood donors in Thailand.

**Fig. 3:**
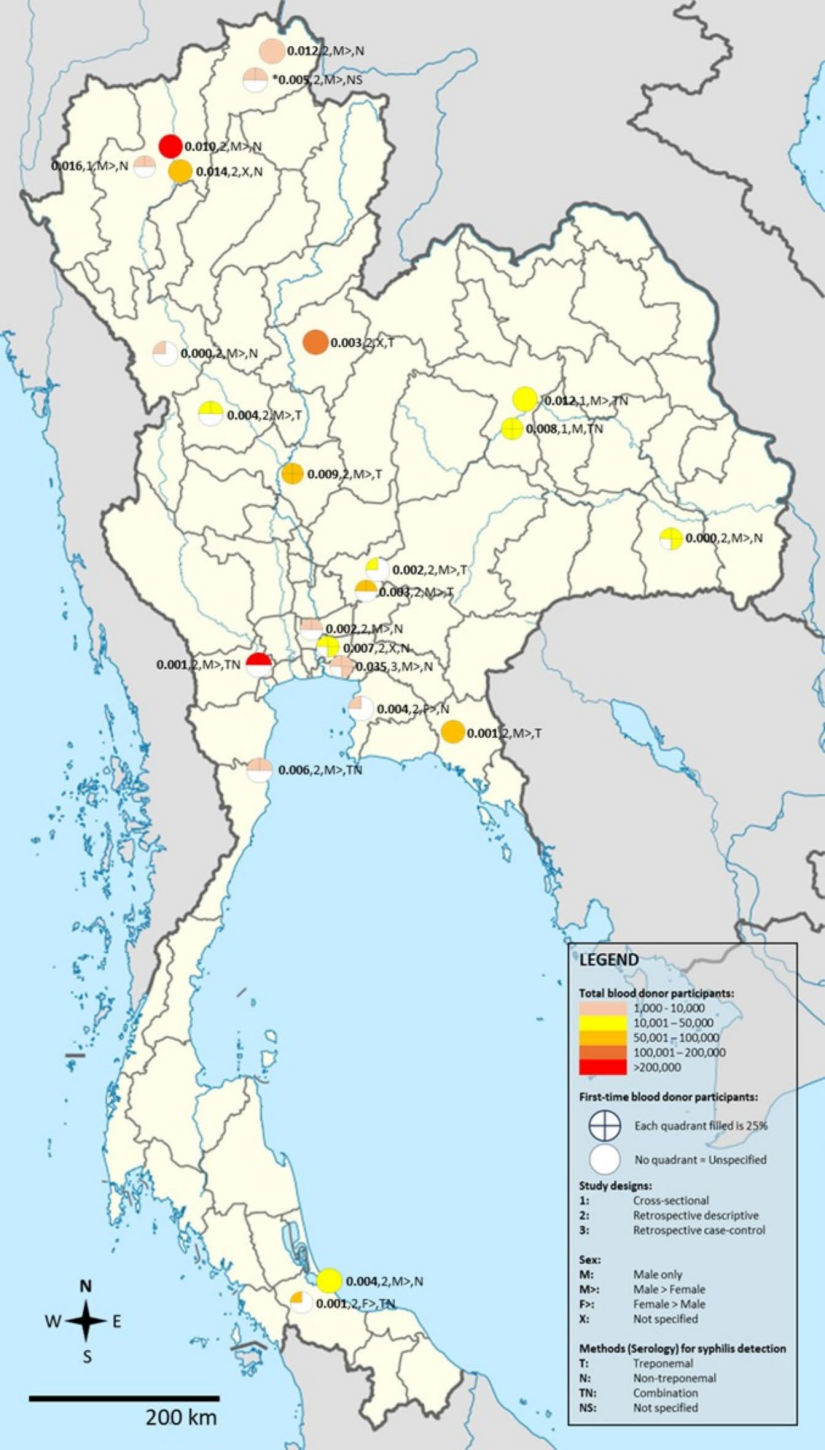
Geographic distribution of the proportion of syphilis in Thailand. All studies had participants in the age range of 16 to 70 years. *All studies had 100% Thai nationality as participants except for Meepradit et al., 2022 in Mae Sai Hospital, Chiang Rai province (Thai: 4,873; 93.4%; Myanma/Burmese: 344; 6.6%). The template of the map was available at https://commons.m.wikimedia.org/wiki/File:Thailand_location_map.svg. This map template is licensed under the Creative Commons Attribution 3.0 Unported license. Permission is granted to copy, distribute and/or modify this document under the terms of the GNU Free Documentation License, Version 1.2 or any later version published by the Free Software Foundation.

The pooled prevalence of syphilis among blood donors was estimated through a random-effects model based on data from 23 studies involving 1,142,910 blood donors. The meta-analysis results using random-effect models showed that the pooled prevalence of syphilis among blood donors in Thailand was 0.42% (95% CI [0.27%; 0.66%], *I²*: 99.3%, number of infections: 6,173, Fig. 2). The geographic distribution of the proportion of syphilis in Thailand is demonstrated in Fig. 3.

The meta-regression analysis suggested that the high heterogeneity of the prevalence (*I²*: 99.3%) was due to several confounders, including publication years (*P* < 0.0001), study design (*P* = 0.0028), male percentage (*P* < 0.0001), and the proportion of first-time donors (*P* = 0.0189) (Supplementary Table S4). Subgroup analysis revealed that the pooled prevalence of syphilis among blood donors in Thailand was highest in studies published before 2000 (1.26%) and decreased during 2000–2009 (0.94%), 2010–2019 (0.27%), and 2020–2024 (0.20%) (Supplementary Table S5). The bubble plot revealed a significant trend of decreasing prevalence of syphilis among blood donors in Thailand from 1995 to 2024 (Supplementary Fig. 1). A difference in prevalence was also observed, with three cross-sectional studies showing a higher prevalence than retrospective descriptive studies (1.13% vs. 0.32%). The subgroup analysis further revealed that Northern Thailand had the highest pooled prevalence compared to other regions (1.08%), although the number of included studies in each subgroup varied. Using serological (nontreponemal) techniques for the detection of syphilis resulted in the highest pooled prevalence (0.51%) compared to other methods (Supplementary Table S5).

### Association between gender and risk of syphilis

**Fig. 4:**
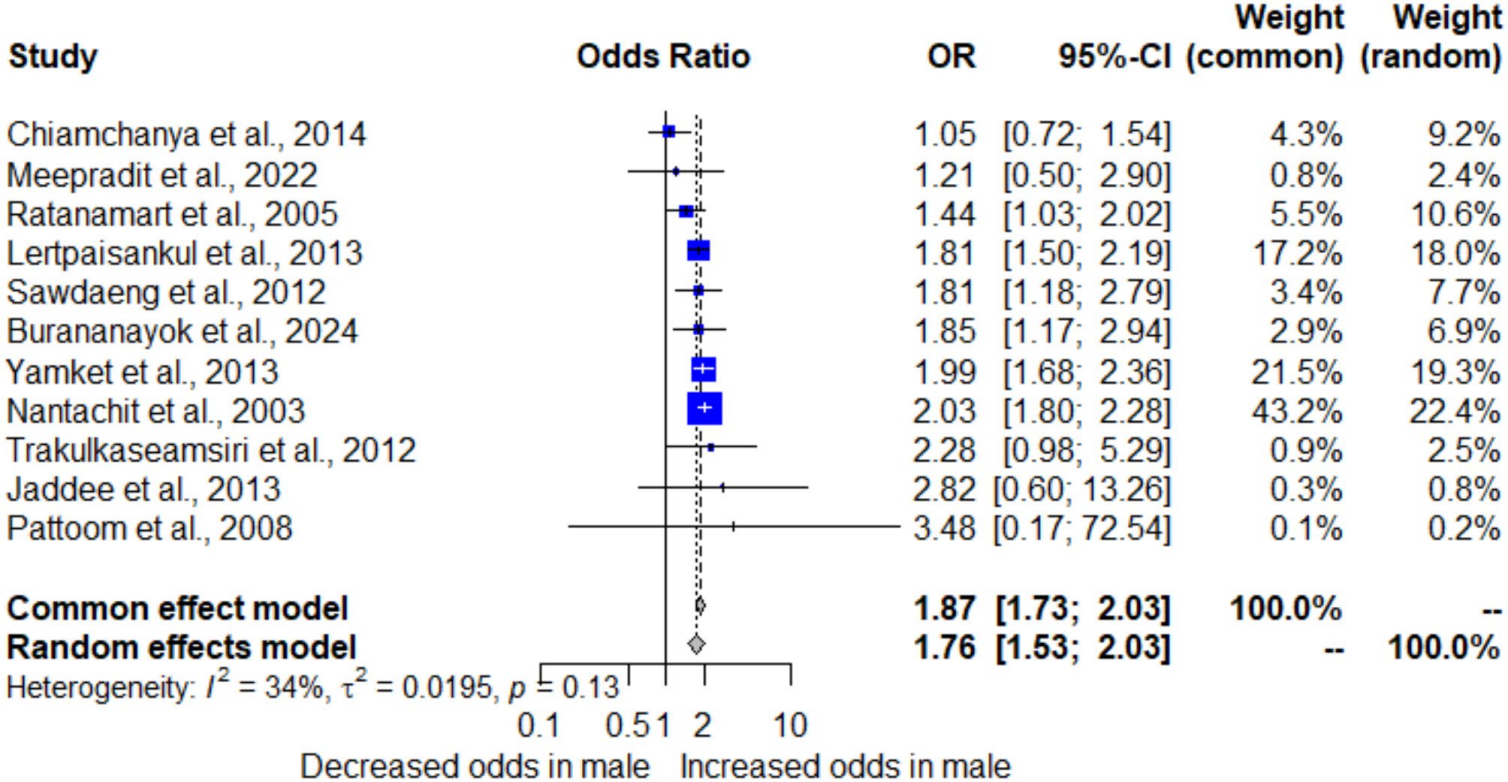
Forest plot showing the OR of syphilis among male blood donors in Thailand.

The association between gender and syphilis in blood donors was estimated using the data from 11 studies that reported the available data for pooling the OR^12,14,15,28,30,32,34–36,39,42^. The meta-analysis results using random-effect models demonstrated that male blood donors were associated with increased risk for syphilis (*P* < 0.0001; pooled OR: 1.76; 95% CI [1.53; 2.03]; *I²*: 34.2%, number of participants: 651,019; Fig. 4). Non-significant heterogeneity was observed in the meta-analysis (*P* = 0.13), with a low degree of heterogeneity. Therefore, meta-regression and subgroup analyses were not conducted.

### Association between frequency of donations and risk of syphilis

**Fig. 5:**
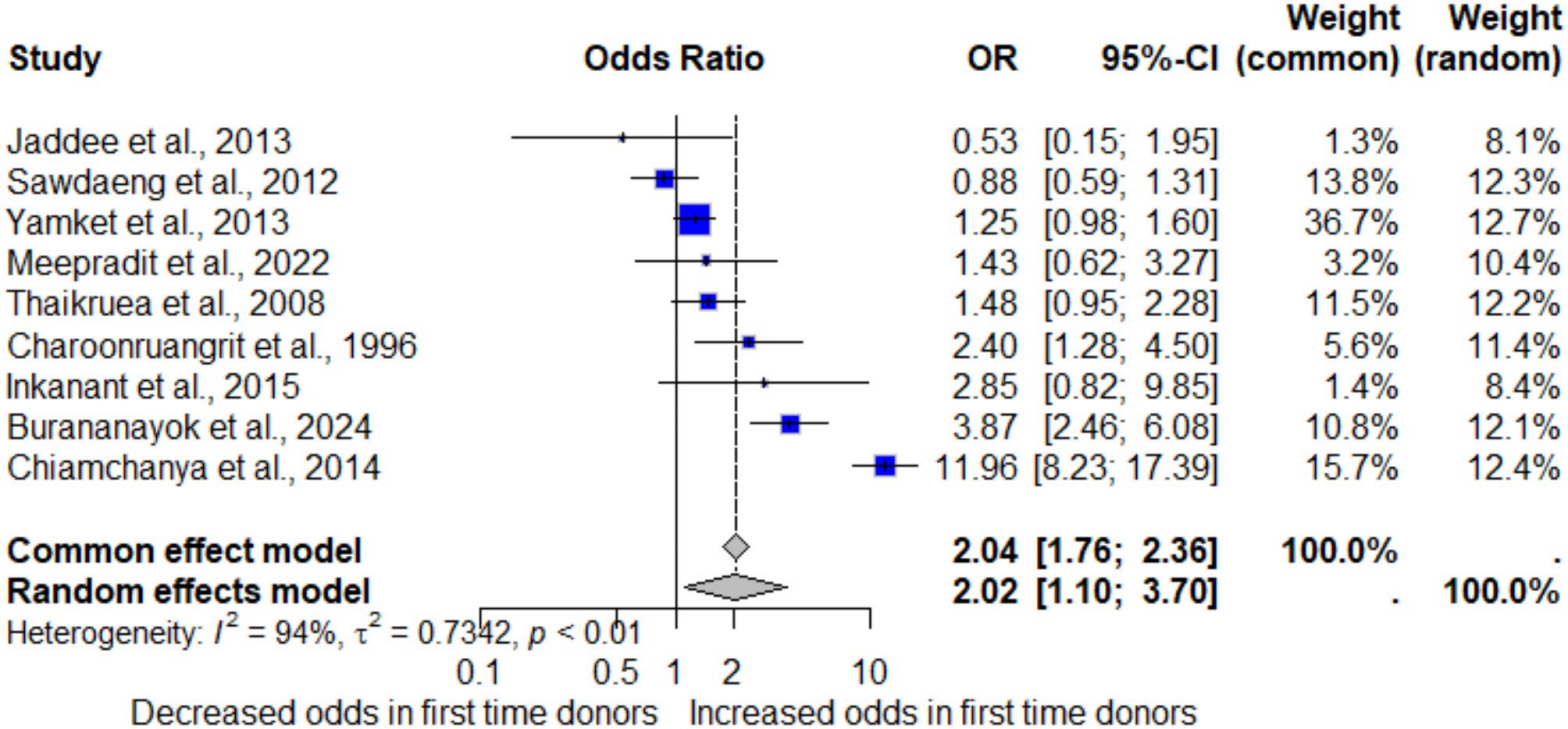
Forest plot showing the odds of syphilis among first-time blood donors in Thailand.

The association between the frequency of donations and the risk of syphilis in blood donors was estimated using the data from nine studies that reported the available data for pooling the OR^12,14,28,30–34,36^. The meta-analysis results using random-effects models demonstrated that first-time donations were associated with an increased risk of syphilis (*P* = 0.023; pooled OR: 2.02; 95% CI [1.10; 3.70]; *I²*: 94.0%, number of participants: 215,245; Fig. 5). The high heterogeneity observed in the meta-analysis may have been caused by the regions of Thailand or methods for syphilis detection, as suggested by the subgroup analysis (Supplementary Table S6).

### Association between age of blood donors and risk of syphilis

The meta-analysis compared the age of blood donors, and the risk of syphilis revealed that participants aged 21–30 years, 31–40 years, and 41–60 years exhibited significantly increased risks of syphilis compared to those aged 17–20 years (OR: 1.63; 3.92; 6.91). Similarly, participants aged 31–40 years and 41–60 years showed significantly higher syphilis risk relative to the 21–30 age group (OR: 2.50; 4.31). Interestingly, participants aged 41–60 years displayed a significantly increased risk of syphilis in comparison to those aged 31–40 years (OR: 1.83, Supplementary Table S7; Supplementary File 1).

### Sensitivity analysis

For the meta-analysis of the pooled prevalence, the meta-analysis results using fixed-effects models demonstrated that the pooled prevalence of syphilis among blood donors in Thailand was 0.54% (95% CI [0.53%; 0.55%], *I²*: 99.3%, Fig. 2). For the association between gender and risk for syphilis, the meta-analysis results using fixed-effects models demonstrated that male blood donors were associated with increased risk for syphilis (*P* < 0.0001; pooled OR: 1.87; 95% CI [1.73; 2.03], Fig. 4). Influential analysis using the random-effects model revealed that after each study was removed and the meta-analysis rerun, the results remained unchanged (*P* < 0.0001, Supplementary File 2). For the association between frequency of blood donations and risk for syphilis, the meta-analysis results using fixed-effects models demonstrated similar findings in which first-time donations were associated with increased risk for syphilis (*P* < 0.0001; pooled OR: 2.04; 95% CI [1.76; 2.36], Fig. 5). Influential analysis using the random-effects model revealed that after each study was removed and the meta-analysis rerun, the meta-analysis results were changed by some studies^30,31^ (*P* < 0.05, Supplementary File 3). The removal of these studies led to a slight increase in the pooled effect size, suggesting that the lower male percentage in these studies may have contributed to a lower overall risk estimate in the original analysis, as demonstrated by Burananayok et al.^30^.

### Publication bias

In the meta-analysis of the pooled prevalence, the funnel plot showed an asymmetrical distribution of prevalence estimates among the included studies relative to the central line (Supplementary Fig. 2). Additionally, Egger’s test revealed significant funnel plot asymmetry (*P* = 0.03), indicating the presence of publication bias in the meta-analysis of the pooled prevalence. For the association between gender and the risk of syphilis, the funnel plot showed an asymmetrical distribution of ORs among the included studies relative to the central line (Supplementary Fig. 3). Additionally, Egger’s test revealed a funnel plot symmetry (*P* = 0.32), indicating the absence of publication bias in the meta-analysis of the OR. For the associations between the frequency of blood donations and the risk of syphilis, as well as between the age of participants and the risk of syphilis, funnel plots were not generated because the number of included studies was less than 10.

## Discussion

The meta-analysis revealed a significant downward trend in the prevalence of syphilis among blood donors in Thailand from 1995 to 2024. Male blood donors were found to be at a higher risk of syphilis infection compared to female donors. First-time donors also exhibited a significantly higher risk of testing positive for syphilis compared to repeat donors. Additionally, an increasing age of donors was strongly associated with an elevated risk of syphilis infection. These findings underscore the importance of targeted interventions, such as enhanced screening and education programs, to mitigate syphilis risk among high-risk donor groups.

The pooled prevalence of syphilis among blood donors decreased from 1.3% in studies published before 2000 to 0.2% in studies published between 2020 and 2024. This decline could be attributed to improved public health interventions, including better screening and prevention programs and increased awareness of STIs. The decline in syphilis prevalence among blood donors may appear to contrast with the rising incidence rates reported in the general population, particularly among specific demographics such as the youth^44^. This apparent contradiction can be explained by the effectiveness of targeted public health strategies in reducing syphilis transmission within the screened blood donor population. For example, rigorous deferral policies and enhanced screening measures have likely contributed to the observed decrease among donors. Conversely, the general population, especially high-risk groups such as the youth and men who have sex with men (MSM)^44^, has experienced an increase in incidence, partly due to broader social and behavioral changes and varying levels of access to prevention and care.

The decline in syphilis infections among blood donors may align with evidence of decreasing prevalence over time in the general population, as reported by a previous meta-analysis^45^. Another meta-analysis that included studies in the Middle East and North Africa revealed a very low prevalence estimate of syphilis among blood donors (0.004%). Still, the prevalence was high among MSM and transgender people (22.52%)^46^. The Ministry of Public Health of Thailand reported a decreasing incidence of syphilis in Thailand between 1985 and 2005. However, the prevalence of syphilis rose again during the 2000–2009 to 2010–2019 periods^47^. The observed decrease in syphilis infections in Thailand, as indicated by the meta-regression analysis, may be attributed to stricter donor selection criteria and more rigorous deferral policies. However, the disruption of syphilis testing during the coronavirus disease 2019 (COVID-19) pandemic could have led to an underestimation of syphilis prevalence in the country. The decline in syphilis cases might also be linked to changes in blood donor demographics across the pre-COVID-19, COVID-19, and post-COVID-19 periods^30^, which has influenced the regulatory screening of blood donations for syphilis. Another possible factor could be the reduced duration of active syphilis infection within the population^48^. Additionally, increased condom use for the prevention of STIs may further explain the decreased prevalence^49^. In addition to syphilis infections, Sinka’s review article reported a modest decline in the age-standardized rate of STIs for almost 30 years from 1990 to 2019^50^. The decrease aligns with global trends of declining syphilis prevalence, particularly in regions where public health strategies have focused on early detection and treatment of STIs.

The meta-analysis findings indicated that male blood donors were at a higher risk of syphilis infection compared to female donors. The pooled OR (OR: 1.76, 95% CI [1.53; 2.03]) suggested a statistically significant association between male gender and increased risk of syphilis. This could be due to higher rates of risky sexual behaviors among men, including multiple sexual partners and inconsistent condom use, which are known risk factors for syphilis transmission. The cultural and social factors that influence male sexual behavior may also play a role in this increased risk. Additionally, the higher prevalence of syphilis among men might reflect broader trends observed in other studies, where men, particularly MSM, have been disproportionately affected by syphilis and other STIs. The meta-analysis results align with a retrospective analysis reporting an increase in syphilis prevalence among males and a decrease among females from 2018 to 2022^30^, as well as another retrospective study showing that syphilis cases predominantly occurred in younger MSM^51^. Factors contributing to the higher incidence among MSM include living with HIV, engaging in condomless anal intercourse, infrequent STI screenings, use of pre-exposure prophylaxis for HIV prevention, having multiple sexual partners, and involvement in the sex trade^52,53^.

The analysis indicates that first-time blood donors are at a significantly higher risk of testing positive for syphilis compared to repeat donors, with a pooled OR of 2.02 (95% CI [1.10; 3.70]). This finding aligns with the previous meta-analysis that suggests first-time donors and replacement donors were more likely to be infected with transfusion transmissible infections than repeat donors^45^. In addition, the meta-analysis results are consistent with a recent study that demonstrated a doubled prevalence of syphilis among first-time donors compared to repeat donors^30^. Repeat donors often undergo regular screening, which may lead to earlier detection and treatment of infections, thus reducing the prevalence of syphilis in this group. Additionally, first-time donors may represent a more heterogeneous population with less strict adherence to donation eligibility criteria, further contributing to the higher observed prevalence. The results highlight the need for targeted screening and education programs for first-time donors to mitigate this risk.

The meta-analysis also shows a strong association between increasing age and the risk of syphilis infection among blood donors. Participants aged 41–60 had the highest risk, with a nearly sevenfold increase compared to those aged 17–20 (OR: 6.91, 95% CI [3.23; 14.76]). The risk also progressively increased with each age group, indicating that older donors are at higher risk of syphilis. This trend could be due to cumulative exposure over time, as older individuals may have had more opportunities to engage in high-risk behaviors. Additionally, the higher prevalence of syphilis in older age groups could reflect historical infection rates, as older individuals were exposed to the infection during periods when syphilis was more prevalent. These findings underscore the importance of continuous education and screening for older adults, who may not perceive themselves as being at risk for STIs. The meta-analysis findings align with a retrospective analysis reporting higher syphilis prevalence among individuals aged 17–40 years and those over 51 years, though it noted a decline in the 41–50-year-old group^30^, contrasting with the results of the present meta-analysis. Similarly, studies and national surveillance data from China observed higher syphilis rates among older adults, particularly those over 45 years, with the highest incidence seen in individuals aged 60 years and older^7,54^.

This study has several limitations that should be considered when interpreting the results. The high level of heterogeneity (*I²* > 90%) may affect the generalizability of the findings. Additionally, the presence of publication bias, indicated by an asymmetrical funnel plot and significant Egger’s test, suggests that studies with lower prevalence estimates may be underreported, potentially leading to an overestimation of syphilis prevalence and risk. Moreover, regional and temporal differences in syphilis prevalence were observed, but the limited number of studies from specific regions and periods may have reduced the robustness of these findings. Furthermore, the analysis was constrained by the availability of data on specific covariates, such as sexual behavior and socioeconomic status, which were not consistently reported across studies. Lastly, the findings are specific to blood donors in Thailand and may not be generalizable to other populations or regions.

The findings of this study highlight the need to strengthen existing national screening policies and public health strategies to further enhance blood safety in Thailand. Current national policies emphasize rigorous screening of all blood donations and donor selection based on low-risk criteria. Building on these measures, targeted interventions could include refining donor screening questionnaires to capture recent high-risk behaviors, improving education campaigns tailored to high-risk groups, such as male and first-time donors, and expanding outreach to older donor populations. Moreover, incorporating advanced syphilis screening technologies, such as nucleic acid testing (NAT), could help detect early or asymptomatic infections, thereby reducing the risk of transmission. Regular evaluation and adaptation of screening protocols in line with epidemiological trends would ensure that Thailand’s blood donation system remains safe and responsive to emerging challenges. By addressing these gaps, the country can enhance its efforts to safeguard the blood supply while reducing the burden of syphilis among high-risk donor groups.

## Conclusion

This systematic review and meta-analysis of 23 studies involving over 1.1 million blood donors in Thailand revealed a pooled syphilis prevalence of 0.42%, with a decreasing trend over time. However, male and first-time donors, as well as older age groups, remain at higher risk. To enhance blood safety, targeted interventions are needed, including refining donor screening questionnaires, expanding educational campaigns for high-risk groups, and incorporating advanced screening technologies. Strengthening and regularly updating national screening policies will ensure the effective mitigation of syphilis transmission among blood donors in Thailand.

## Electronic supplementary material

Below is the link to the electronic supplementary material.

Supplementary Material 1

Supplementary Material 2

Supplementary Material 3

Supplementary Material 4

Supplementary Material 5

## Data Availability

All data relating to the present study are available in this manuscript, Table S1, Table S2, Table S3, Supplementary File and Tables, and Supplementary Figure files.

## Abbreviations

CIs: Confidence intervals
COVID-19: Coronavirus disease 2019
HBV: Hepatitis B virus
HCV: Hepatitis C virus
HIV: Human immunodeficiency virus
JBI: The Joanna Briggs Institute
MSM: Men who have sex with men
NAT: Nucleic acid testing
ORs: Odds ratios
PRISMA: Preferred Reporting Items for Systematic Reviews and Meta-Analyses
STIs: Sexually transmitted infections
TCI: Thai Journal Citation Index
TTIs: Transfusion-transmissible infections
WHO: World Health Organization
