# Supplementary material for: A systematic review and meta-analysis of the prevalence and risk of syphilis among blood donors in Thailand
Source: Sci Rep. 2025 Mar 18;15:9316. doi: 10.1038/s41598-025-94332-3 (PMC11920362; doi:10.1038/s41598-025-94332-3)
Supplement: Supplementary file 1 — Supplementary Material 1 [file 41598_2025_94332_MOESM1_ESM.docx]

**Table S1. Search terms and search strategies used in each database**

**General keywords**

(Syphilis OR “Treponema pallidum” OR Chancre OR “Neurosyphilis” OR “Tabes Dorsalis” OR “Spirochaetales Infections” OR “Spirochaetales” OR “Treponemal Infections”) AND (“Blood Donors” OR “Blood Donor” OR Donor OR Donors) AND (Asia OR Asian OR “Southeast Asia” OR “Southeast Asian” OR Thailand OR Siam)

PubMed 20 June 2024

| No. | Key concept | Search terms | Results |
| --- | --- | --- | --- |
| 1. | Syphilis | Syphilis[All Fields] OR “Treponema pallidum”[All Fields] OR Chancre[All Fields] OR “Neurosyphilis”[All Fields] OR “Tabes Dorsalis”[All Fields] OR Syphilis[MeSH Terms] OR “Treponema pallidum”[MeSH Terms] OR Chancre[MeSH Terms] OR “Neurosyphilis”[MeSH Terms] OR “Tabes Dorsalis”[MeSH Terms] | 43,763 |
| 2. | Blood donor | “Blood Donors”[All Fields] OR “Blood Donor”[All Fields] OR Donor[All Fields] OR Donors[All Fields] OR “Blood Donors”[MeSH Terms] OR “Blood Donor”[MeSH Terms] OR Donor[MeSH Terms] OR Donors[MeSH Terms] | 406,779 |
| 3. | Thailand | Asia[All Fields] OR Asian[All Fields] OR “Southeast Asia” [All Fields] OR “Southeast Asian” [All Fields] OR Thailand[All Fields] OR Siam[All Fields] OR Asia[MeSH Terms] OR Asian[MeSH Terms] OR “Southeast Asia” [MeSH Terms] OR “Southeast Asian” [MeSH Terms] OR Thailand[MeSH Terms] OR Siam[MeSH Terms] | 1,404,852 |
| 4. | #1 AND #2 AND #3 AND #4 | (Syphilis[All Fields] OR “Treponema pallidum”[All Fields] OR Chancre[All Fields] OR “Neurosyphilis”[All Fields] OR “Tabes Dorsalis”[All Fields] OR Syphilis[MeSH Terms] OR “Treponema pallidum”[MeSH Terms] OR Chancre[MeSH Terms] OR “Neurosyphilis”[MeSH Terms] OR “Tabes Dorsalis”[MeSH Terms]) AND (“Blood Donors”[All Fields] OR “Blood Donor”[All Fields] OR Donor[All Fields] OR Donors[All Fields] OR “Blood Donors”[MeSH Terms] OR “Blood Donor”[MeSH Terms] OR Donor[MeSH Terms] OR Donors[MeSH Terms]) AND (Asia[All Fields] OR Asian[All Fields] OR “Southeast Asia” [All Fields] OR “Southeast Asian” [All Fields] OR Thailand[All Fields] OR Siam[All Fields] OR Asia[MeSH Terms] OR Asian[MeSH Terms] OR “Southeast Asia” [MeSH Terms] OR “Southeast Asian” [MeSH Terms] OR Thailand[MeSH Terms] OR Siam[MeSH Terms]) | 188 |

Embase 20 June 2024

| No. | Key concept | Search terms | Results |
| --- | --- | --- | --- |
| 1. | Syphilis | Syphilis:ti,ab,kw,de OR “Treponema pallidum”:ti,ab,kw,de OR Chancre:ti,ab,kw,de OR “Neurosyphilis”:ti,ab,kw,de OR “Tabes Dorsalis”:ti,ab,kw,de OR Syphilis/exp OR “Treponema pallidum”/exp OR Chancre/exp OR “Neurosyphilis”/exp OR “Tabes Dorsalis”/exp | 59806 |
| 2. | Blood donor | “Blood Donors”:ti,ab,kw,de OR “Blood Donor”:ti,ab,kw,de OR Donor:ti,ab,kw,de OR Donors:ti,ab,kw,de OR “Blood Donors”/exp OR “Blood Donor”/exp OR Donor/exp OR Donors/exp | 589,055 |
| 3. | Thailand | Asia:ti,ab,kw,de OR Asian:ti,ab,kw,de OR “Southeast Asia”:ti,ab,kw,de OR “Southeast Asian”:ti,ab,kw,de OR Thailand:ti,ab,kw,de OR Siam:ti,ab,kw,de OR Asia/exp OR Asian/exp OR “Southeast Asia”/exp OR “Southeast Asian”/exp OR Thailand/exp OR Siam/exp | 1,815,526 |
| 4. | 1 AND 2 AND 3 |  | 482 |

Scopus 20 June 2024

| No. | Key concept | Search terms | Results |
| --- | --- | --- | --- |
| 1. | Syphilis | TITLE-ABS-KEY ( syphilis OR "treponema pallidum" OR chancre OR "neurosyphilis" OR "tabes dorsalis" OR "spirochaetales infections" OR "spirochaetales" OR "treponemal infections" ) | 64,883 |
| 2. | Blood donor | TITLE-ABS-KEY ( "blood donors" OR "blood donor" OR donor OR donors ) | 642,216 |
| 3. | Thailand | TITLE-ABS-KEY ( asia OR asian OR "southeast asia" OR "southeast asian" OR thailand OR siam ) | 853,478 |
| 4. | 1 AND 2 AND 3 | ( TITLE-ABS-KEY ( syphilis OR "Treponema pallidum" OR chancre OR "Neurosyphilis" OR "Tabes Dorsalis" OR "Spirochaetales Infections" OR "Spirochaetales" OR "Treponemal Infections" ) ) AND ( TITLE-ABS-KEY ( "Blood Donors" OR "Blood Donor" OR donor OR donors ) ) AND ( TITLE-ABS-KEY ( asia OR asian OR "Southeast Asia" OR "Southeast Asian" OR thailand OR siam ) ) | 46 |

MEDLINE 20 June 2024

| No. | Key concept | Search terms | Results |
| --- | --- | --- | --- |
| 1. | Syphilis AND Blood donor AND Thailand | (Syphilis OR “Treponema pallidum” OR Chancre OR “Neurosyphilis” OR “Tabes Dorsalis” OR “Spirochaetales Infections” OR “Spirochaetales” OR “Treponemal Infections”) AND (“Blood Donors” OR “Blood Donor” OR Donor OR Donors) AND (Asia OR Asian OR “Southeast Asia” OR “Southeast Asian” OR Thailand OR Siam) | 43 |

Ovid 20 June 2024

| No. | Key concept | Search terms | Results |
| --- | --- | --- | --- |
| 1. | IP-10 AND Malaria | (Syphilis OR “Treponema pallidum” OR Chancre OR “Neurosyphilis” OR “Tabes Dorsalis” OR “Spirochaetales Infections” OR “Spirochaetales” OR “Treponemal Infections”) AND (“Blood Donors” OR “Blood Donor” OR Donor OR Donors) AND (Asia OR Asian OR “Southeast Asia” OR “Southeast Asian” OR Thailand OR Siam) | 154 |

ProQuest 20 June 2024

| No. | Key concept | Search terms | Results |
| --- | --- | --- | --- |
| 1. | Syphilis AND Blood donor AND Thailand | (Syphilis OR “Treponema pallidum” OR Chancre OR “Neurosyphilis” OR “Tabes Dorsalis” OR “Spirochaetales Infections” OR “Spirochaetales” OR “Treponemal Infections”) AND (“Blood Donors” OR “Blood Donor” OR Donor OR Donors) AND (Asia OR Asian OR “Southeast Asia” OR “Southeast Asian” OR Thailand OR Siam) | 1,317 |

Google Scholar 20 June 2024

| No. | Key concept | Search terms | Results |
| --- | --- | --- | --- |
| 1. | Syphilis AND Blood donor AND Thailand | Syphilis AND Blood Donors AND Thailand | The first 200 articles |

TCI 20 June 2024

| No. | Key concept | Search terms | Results |
| --- | --- | --- | --- |
| 1. | Syphilis AND Blood donor AND Thailand | ซิฟิลิส OR Syphilis | 125 (related 19) |
