# Supplementary material for: A systematic review and meta-analysis of the prevalence and risk of syphilis among blood donors in Thailand
Source: Sci Rep. 2025 Mar 18;15:9316. doi: 10.1038/s41598-025-94332-3 (PMC11920362; doi:10.1038/s41598-025-94332-3)
Supplement: Supplementary file 4 — Supplementary Material 4 [file 41598_2025_94332_MOESM4_ESM.docx]

**Supplementary Tables and Files**

**Supplementary Table S4. Meta-regression analysis of the pooled prevalence**

| **Covariates** | **τ** | **Test for residual heterogeneity, *P* value** | **Residual heterogeneity *I^2^* (%)** | **Test of moderators, *P* value** | **Number of studies** |
| --- | --- | --- | --- | --- | --- |
| Publication years | 0.5839 | < 0.0001 | 99.15 | < 0.0001 | 23 |
| Study design | 0.7487 | < 0.0001 | 99.39 | 0.0028 | 23 |
| Regions of Thailand | 0.8496 | < 0.0001 | 99.31 | 0.2551 | 23 |
| Male percentage | 0.6266 | < 0.0001 | 98.77 | < 0.0001 | 20 |
| First-time donation | 0.9575 | < 0.0001 | 98.70 | 0.0189 | 16 |
| Onsite donation percentage | 0.4201 | < 0.0001 | 96.01 | 0.6990 | 9 |
| Method for syphilis detection | 1.0949 | < 0.0001 | 95.51 | 0.7911 | 23 |

**Supplementary Table S5. Prevalence of syphilis stratified by characteristics of the study population**

| **Parameters** | **Subgroups** | **Pooled proportion [95% CI] %** | ***I^2^* (%)** | **Number of studies** |
| --- | --- | --- | --- | --- |
| Overall |  | 0.43 [0.27; 0.68] | 99.5% | 23 |
| Publication year |  |  |  |  |
|  | Before 2000 | 1.26 [0.85; 1.88] | 96.0 | 6 |
|  | 2000–2009 | 0.94 [0.91; 0.97] | 96.6 | 4 |
|  | 2010–2019 | 0.27 [0.15; 0.51] | 99.1 | 8 |
|  | 2020–2024 | 0.20 [0.13; 0.32] | 96.6 | 5 |
| Study design |  |  |  |  |
|  | Retrospective descriptive study | 0.32 [0.21; 0.50] | 99.3 | 19 |
|  | Cross-sectional study | 1.13 [1.02; 1.26] | 89.1 | 3 |
|  | Retrospective study, case-control | 3.54 [2.68; 4.66] | N/A | 1 |
| Regions of Thailand |  |  |  |  |
|  | Northern Thailand | 1.08 [0.77; 1.53] | 96.1 | 5 |
|  | Central Thailand | 0.47 [0.17; 1.30] | 98.8 | 5 |
|  | Lower northern Thailand | 0.31 [0.12; 0.81] | 99.0 | 4 |
|  | Northeastern Thailand | 0.36 [0.07; 1.84] | 98.0 | 3 |
|  | Southern Thailand | 0.23 [0.09; 0.56] | 98.9 | 2 |
|  | Eastern Thailand | 0.23 [0.10; 0.52] | 96.3 | 2 |
|  | Western Thailand | 0.25 [0.09; 0.76] | 99.1 | 2 |
| Method for syphilis |  |  |  |  |
|  | Serology (nontreponemal) | 0.51 [0.23; 1.13] | 98.0 | 11 |
|  | Serology (treponemal) | 0.31 [0.19; 0.52] | 99.1 | 6 |
|  | Serology (treponemal/nontreponemal) | 0.38 [0.16; 0.89] | 99.5 | 5 |
|  | Not specified | 0.48 [0.32; 0.71] | N/A | 1 |

N/A, not assessed; CI, confidence interval

**Supplementary Table S6. Association between first-time blood donors and risk of syphilis**

**Subgroup analysis**

| **Pooled odds ratio** |  | **95% CI** | ***I^2^* (%)** | **Number of studies** |
| --- | --- | --- | --- | --- |
| Overall | *P* value = 0.0225 | 2.0226 [1.1044; 3.7041] | 94.0 | 9 |
| Publication year |  |  |  |  |
|  | 2010–2019 | 1.6395 [1.1381; 2.3618] | 68.1 | 4 |
|  | 2020–2024 | 1.6869 [1.1217; 2.5367] | 0.0 | 2 |
| Study design |  |  |  |  |
|  | Retrospective descriptive study | 1.6417 [1.2593; 2.1401] | 51.0 | 6 |
| Regions of Thailand |  |  |  |  |
|  | Lower northern Thailand | 1.8145 [1.1820; 2.7853] | N/A | 1 |
|  | Southern Thailand | 1.8511 [1.1671; 2.9358] | N/A | 1 |
|  | Northern Thailand | 1.2075 [0.5034; 2.8965] | N/A | 1 |
|  | Central Thailand | 1.0543 [0.7232; 1.5369] | N/A | 1 |
|  | Northeastern Thailand | 2.8153 [0.5977; 13.2603] | N/A | 1 |
|  | Western Thailand | 1.9900 [1.6798; 2.3575] | N/A | 1 |
| Method for syphilis |  |  |  |  |
|  | Serology (nontreponemal) | 2.8153 [0.5977; 13.2603] | N/A | 1 |
|  | Serology (treponemal) | 1.3694 [0.8046; 2.3304] | 71.2 | 2 |
|  | Serology (treponemal/nontreponemal) | 1.9730 [1.6828; 2.3131] | 0.0 | 2 |
|  | Not specified | 1.2075 [0.5034; 2.8965] | N/A | 1 |

N/A, not assessed

**Supplementary Table S7. Risk of syphilis among blood donors in Thailand between specific age groups**

| **Age range (comparator)** | **Age groups,**  **odds ratio** [**CI**]***** | | |
| --- | --- | --- | --- |
|  | **21-30 years** | **31-40 years** | **41-60 years** |
| **17–20 years** | 1.63 [1.05; 2.52] | 3.92 [1.86; 8.28] | 6.91 [3.23; 14.76] |
| **21-30 years** |  | 2.50 [1.52; 4.10] | 4.31 [2.18; 8.50] |
| **31-40 years** |  |  | 1.83 [1.37; 2.46] |

*All results are statistically significant. Odds ratio > 1 indicates the fold increased risk of having syphilis.

**Supplementary File 1.** **Association between age of blood donors and risk of syphilis**

**5.1. 17-20 vs. 21-30 years**

Number of studies: k = 8

Number of observations: o = 173297 (o.e = 90319, o.c = 82978)

Number of events: e = 343

OR 95%-CI z p-value

Common effect model 1.5162 [1.2141; 1.8936] 3.67 0.0002

Random effects model 1.6297 [1.0546; 2.5183] 2.20 0.0278

Quantifying heterogeneity:

τ^2^ = 0.1860 [0.0038; 3.5855]; τ = 0.4313 [0.0619; 1.8935]

*I²* = 57.5% [6.6%; 80.6%]; H = 1.53 [1.03; 2.27]

Test of heterogeneity:

Q d.f. p-value

16.46 7 0.0213

Details on meta-analytical method:

- Inverse variance method

- Restricted maximum-likelihood estimator for τ^2^

- Q-Profile method for confidence interval of τ^2^ and τ

- Continuity correction of 0.5 in studies with zero cell frequencies

**5.2. 17-20 vs. 31-40 years**

Number of studies: k = 9

Number of observations: o = 142967 (o.e = 59989, o.c = 82978)

Number of events: e = 547

OR 95%-CI z p-value

Common effect model 4.9603 [4.0725; 6.0417] 15.92 < 0.0001

Random effects model 3.9214 [1.8572; 8.2799] 3.58 0.0003

Quantifying heterogeneity:

τ^2^ = 0.9083 [0.2385; 4.6834]; τ = 0.9530 [0.4884; 2.1641]

*I²* = 80.6% [64.0%; 89.5%]; H = 2.27 [1.67; 3.09]

Test of heterogeneity:

Q d.f. p-value

41.23 8 < 0.0001

Details on meta-analytical method:

- Inverse variance method

- Restricted maximum-likelihood estimator for τ^2^

- Q-Profile method for confidence interval of τ^2^ and τ

- Continuity correction of 0.5 in studies with zero cell frequencies

**5.3. 17-20 vs. 41-60 years**

Number of studies: k = 9

Number of observations: o = 135168 (o.e = 52190, o.c = 82978)

Number of events: e = 757

OR 95%-CI z p-value

Common effect model 8.7887 [7.2376; 10.6721] 21.94 < 0.0001

Random effects model 6.9080 [3.2335; 14.7585] 4.99 < 0.0001

Quantifying heterogeneity:

τ^2^ = 0.9560 [0.2646; 3.5584]; τ = 0.9777 [0.5144; 1.8864]

*I²* = 87.3% [78.0%; 92.7%]; H = 2.81 [2.13; 3.69]

Test of heterogeneity:

Q d.f. p-value

62.99 8 < 0.0001

Details on meta-analytical method:

- Inverse variance method

- Restricted maximum-likelihood estimator for τ^2^

- Q-Profile method for confidence interval of τ^2^ and τ

- Continuity correction of 0.5 in studies with zero cell frequencies

**5.4. 21-30 vs. 31-40 years**

Number of studies: k = 9

Number of observations: o = 150308 (o.e = 59989, o.c = 90319)

Number of events: e = 610

OR 95%-CI z p-value

Common effect model 3.2083 [2.6935; 3.8214] 13.06 < 0.0001

Random effects model 2.4976 [1.5248; 4.0909] 3.64 0.0003

Quantifying heterogeneity:

τ^2^ = 0.3774 [0.0919; 1.4647]; τ = 0.6143 [0.3032; 1.2103]

*I²* = 82.2% [67.4%; 90.3%]; H = 2.37 [1.75; 3.20]

Test of heterogeneity:

Q d.f. p-value

44.90 8 < 0.0001

Details on meta-analytical method:

- Inverse variance method

- Restricted maximum-likelihood estimator for τ^2^

- Q-Profile method for confidence interval of τ^2^ and τ

- Continuity correction of 0.5 in studies with zero cell frequencies

**5.5. 21-30 vs. 41-60 years**

Number of studies: k = 9

Number of observations: o = 142509 (o.e = 52190, o.c = 90319)

Number of events: e = 820

OR 95%-CI z p-value

Common effect model 5.6760 [4.7792; 6.7412] 19.79 < 0.0001

Random effects model 4.3063 [2.1819; 8.4991] 4.21 < 0.0001

Quantifying heterogeneity:

τ^2^ = 0.8315 [0.2751; 3.8820]; τ = 0.9118 [0.5245; 1.9703]

*I²* = 90.8% [84.8%; 94.4%]; H = 3.29 [2.56; 4.23]

Test of heterogeneity:

Q d.f. p-value

86.77 8 < 0.0001

Details on meta-analytical method:

- Inverse variance method

- Restricted maximum-likelihood estimator for τ^2^

- Q-Profile method for confidence interval of τ^2^ and τ

- Continuity correction of 0.5 in studies with zero cell frequencies

**5.6. 31-40 vs. 41-60 years**

Number of studies: k = 9

Number of observations: o = 112179 (o.e = 52190, o.c = 59989)

Number of events: e = 1024

OR 95%-CI z p-value

Common effect model 1.9746 [1.7338; 2.2487] 10.26 < 0.0001

Random effects model 1.8348 [1.3709; 2.4557] 4.08 < 0.0001

Quantifying heterogeneity:

τ^2^ = 0.1002 [0.0214; 1.7649]; τ = 0.3165 [0.1461; 1.3285]

*I²* = 71.2% [43.0%; 85.4%]; H = 1.86 [1.32; 2.62]

Test of heterogeneity:

Q d.f. p-value

27.75 8 0.0005

Details on meta-analytical method:

- Inverse variance method

- Restricted maximum-likelihood estimator for τ^2^

- Q-Profile method for confidence interval of τ^2^ and τ

**Supplementary File 2. Gender and risk of syphilis**

**Influential analysis (random effects model)**

OR 95%-CI p-value τ^2^ τ

Omitting Burananayok et al., 2024 1.7443 [1.4902; 2.0417] < 0.0001 0.0248 0.1575

Omitting Chiamchanya et al., 2014 1.9234 [1.7750; 2.0843] < 0.0001 0.0000 0.0000

Omitting Jaddee et al., 2013 1.7529 [1.5185; 2.0236] < 0.0001 0.0204 0.1427

Omitting Lertpaisankul et al., 2013 1.7303 [1.4481; 2.0675] < 0.0001 0.0310 0.1761

Omitting Meepradit et al., 2022 1.7789 [1.5440; 2.0496] < 0.0001 0.0189 0.1375

Omitting Nantachit et al., 2003 1.6889 [1.4324; 1.9913] < 0.0001 0.0218 0.1475

Omitting Pattoom et al., 2008 1.7584 [1.5258; 2.0266] < 0.0001 0.0198 0.1406

Omitting Ratanamart et al., 2005 1.8117 [1.5726; 2.0871] < 0.0001 0.0162 0.1272

Omitting Sawdaeng et al., 2012 1.7457 [1.4890; 2.0468] < 0.0001 0.0253 0.1590

Omitting Trakulkaseamsiri et al., 2012 1.7448 [1.5055; 2.0221] < 0.0001 0.0217 0.1472

Omitting Yamket et al., 2013 1.7002 [1.4337; 2.0162] < 0.0001 0.0262 0.1619

Pooled estimate 1.7617 [1.5298; 2.0287] < 0.0001 0.0195 0.1395

*I²*

Omitting Burananayok et al., 2024 40.7%

Omitting Chiamchanya et al., 2014 0.0%

Omitting Jaddee et al., 2013 39.7%

Omitting Lertpaisankul et al., 2013 40.2%

Omitting Meepradit et al., 2022 36.7%

Omitting Nantachit et al., 2003 26.6%

Omitting Pattoom et al., 2008 40.1%

Omitting Ratanamart et al., 2005 29.1%

Omitting Sawdaeng et al., 2012 40.7%

Omitting Trakulkaseamsiri et al., 2012 39.9%

Omitting Yamket et al., 2013 38.3%

Pooled estimate 34.2%

Details on meta-analytical method:

- Inverse variance method

- Restricted maximum-likelihood estimator for τ^2^

**Supplementary File 3. Association between first-time blood donors and risk of syphilis**

**Influential analysis (random effects model)**

OR 95%-CI p-value τ^2^ τ *I²*

Omitting Burananayok et al., 2024 1.8462 [0.9490; 3.5916] 0.0709 0.7878 0.8876 94.4%

Omitting Charoonruangrit et al., 1996 1.9701 [0.9933; 3.9074] 0.0523 0.8479 0.9208 94.7%

Omitting Chiamchanya et al., 2014 1.5881 [1.0525; 2.3960] 0.0275 0.2395 0.4894 77.8%

Omitting Inkanant et al., 2015 1.9524 [1.0066; 3.7869] 0.0478 0.8148 0.9026 94.7%

Omitting Jaddee et al., 2013 2.2761 [1.2346; 4.1961] 0.0084 0.6840 0.8271 94.6%

Omitting Meepradit et al., 2022 2.0992 [1.0712; 4.1138] 0.0307 0.8236 0.9075 94.7%

Omitting Sawdaeng et al., 2012 2.2758 [1.2000; 4.3160] 0.0118 0.7183 0.8475 93.8%

Omitting Thaikruea et al., 2008 2.1031 [1.0617; 4.1662] 0.0330 0.8371 0.9149 94.7%

Omitting Yamket et al., 2013 2.1598 [1.0980; 4.2485] 0.0257 0.8123 0.9013 93.6%

Pooled estimate 2.0226 [1.1044; 3.7041] 0.0225 0.7342 0.8569 94.0%

Details on meta-analytical method:

- Inverse variance method

- Restricted maximum-likelihood estimator for τ^2^
