# Supplementary material for: A systematic review and meta-analysis of the prevalence and risk of syphilis among blood donors in Thailand
Source: Sci Rep. 2025 Mar 18;15:9316. doi: 10.1038/s41598-025-94332-3 (PMC11920362; doi:10.1038/s41598-025-94332-3)
Supplement: Supplementary file 5 — Supplementary Material 5 [file 41598_2025_94332_MOESM5_ESM.docx]

**Supplementary Figures**

**
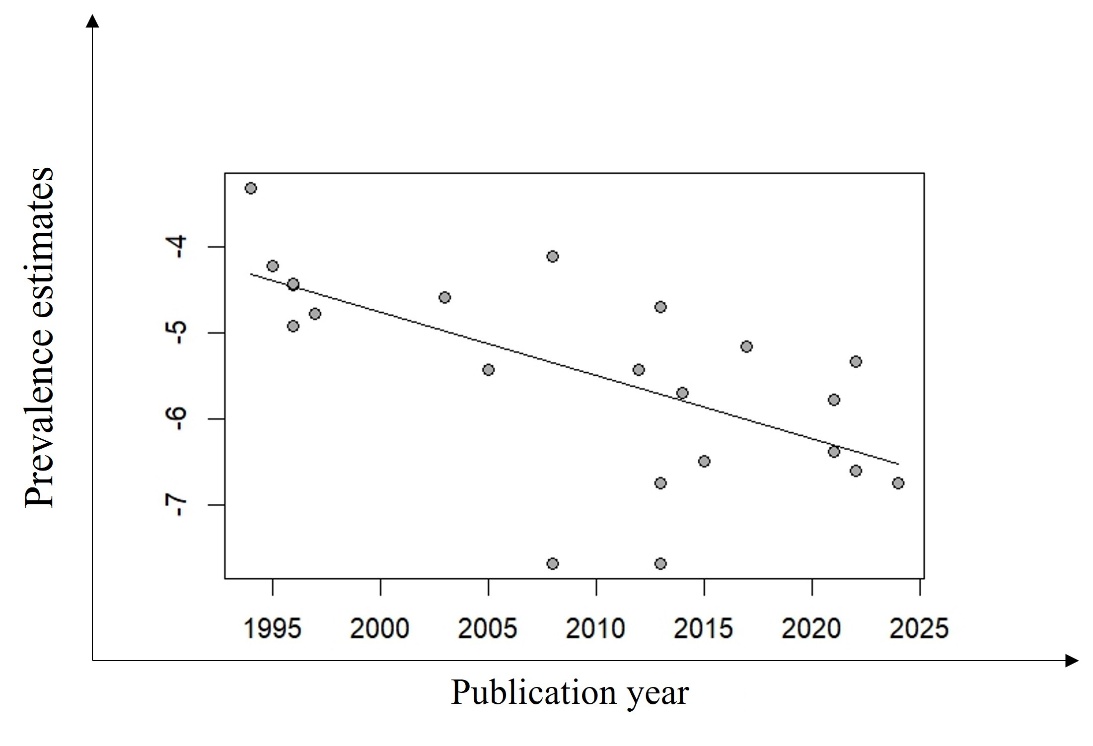
**

**Supplementary Figure 1.** The bubble plot presents the association between publication years and log-prevalence estimates of the outcome. Each bubble represents an individual study, with its position on the x-axis corresponding to the publication year and its position on the y-axis representing the log-prevalence. X-Axis (covariate: publication year) shows the publication year of the studies included in the meta-analysis, ranging from approximately 1995 to 2025. Y-Axis (log-prevalence) displays the log-transformed prevalence estimates reported by the studies. The line of best fit shows the overall trend in the data.

**
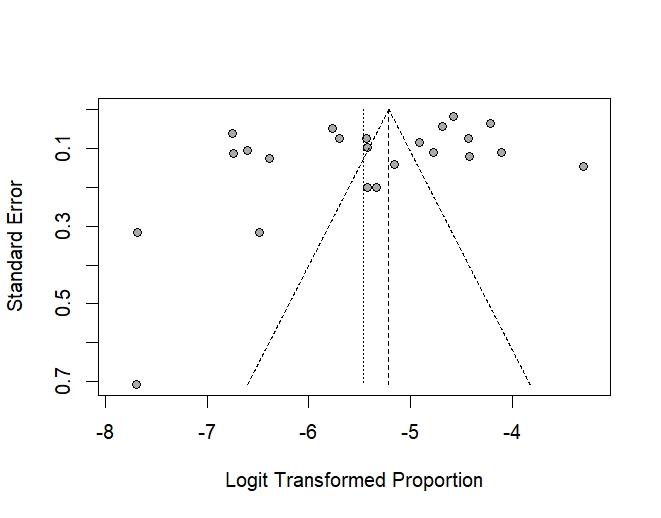
**

**Supplementary Figure 2.** The funnel plot visualizes the potential publication bias in the meta-analysis by plotting the standard error of the logit-transformed proportion estimates against their corresponding values. X-Axis (logit-transformed proportion) represents the logit-transformed proportion estimates from the studies included in the meta-analysis. Y-Axis (standard error) shows the standard error associated with each logit-transformed proportion. The vertical dashed line represents the overall pooled effect estimate.

**
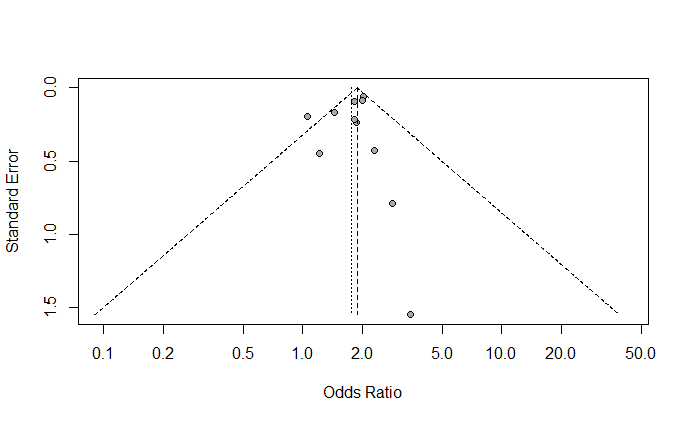
**

**Supplementary Figure 3.** The funnel plot visualizes the potential publication bias in the meta-analysis by plotting the standard error of the odds ratio against their corresponding values. X-Axis represents the odds ratio from the studies included in the meta-analysis. Y-Axis (standard error) shows the standard error associated with each odds ratio. The vertical dashed line represents the overall pooled effect estimate.
